# Development of an Electroactive and Thermo-Reversible Diels–Alder Epoxy Nanocomposite Doped with Carbon Nanotubes

**DOI:** 10.3390/polym15244715

**Published:** 2023-12-15

**Authors:** Isaac Lorero, Álvaro Rodríguez, Mónica Campo, Silvia G. Prolongo

**Affiliations:** 1 Materials Science and Engineering Area, Rey Juan Carlos University, C/Tulipán s/n, 28933 Móstoles, Madrid, Spain; a.rodriguezgonzalez10@alumnos.urjc.es (Á.R.); monica.campo@urjc.es (M.C.); 2 Instituto de Tecnologías Para la Sostenibilidad, Rey Juan Carlos University, C/Tulipán s/n, 28933 Móstoles, Madrid, Spain

**Keywords:** Diels–Alder thermoset, nanocomposites, shape memory, electroactive polymer, CAN

## Abstract

The manufacturing of Diels–Alder (D-A) crosslinked epoxy nanocomposites is an emerging field with several challenges to overcome: the synthesis is complex due to side reactions, the mechanical properties are hindered by the brittleness of these bonds, and the content of carbon nanotubes (CNT) added to achieve electroactivity is much higher than the percolation thresholds of other conventional resins. In this work, we develop nanocomposites with different D-A crosslinking ratios (0, 0.6, and 1.0) and CNT contents (0.1, 0.3, 0.5, 0.7, and 0.9 wt.%), achieving a simplified route and avoiding the use of solvents and side reactions by selecting a two-step curing method (100 °C-6 h + 60 °C-12 h) that generates the thermo-reversible resins. These reversible nanocomposites show ohmic behavior and effective Joule heating, reaching the dissociation temperatures of the D-A bonds. The fully reversible nanocomposites (ratio 1.0) present more homogeneous CNT dispersion compared to the partially reversible nanocomposites (ratio 0.6), showing higher electrical conductivity, as well as higher brittleness. For this study, the nanocomposite with a partially reversible matrix (ratio 0.6) doped with 0.7 CNT wt.% was selected to allow us to study its new smart functionalities and performance due to its reversible network by analyzing self-healing and thermoforming.

## 1. Introduction

Research into covalent adaptable networks (CAN) is a field that has attracted growing interest in recent years. The combination of thermoset characteristics, such as adequate mechanical and thermo-mechanical properties, environmental resistance and wettability, and adhesion to reinforcing fibers [1], together with self-healing and recyclability, through dynamic crosslinks [2,3] enables the manufacturing of a new family of materials that encourage the development of advanced and sustainable solutions for resolving the industrial challenges related to the use of polymer composites. The structures of these polymers can be reorganized through stimulated bond exchanging or disarrangement. The exchange is usually triggered through heating, but other stimuli, such as light or pH changes, can be used depending on the bond chemistry [4]. As a function of whichever mechanism is used to allow this transition, these polymers are classified into the group of associative or dissociative CANs. On one hand, the associative CANs maintain a constant cross-link density during rearrangement via different reactions such as transesterification catalyst [5,6], or disulfide bonding [7], and imine exchanges [8]. On the other hand, the dissociative CANs show bond breakage, leading to network fragmentation and even flow. After the exchange activation, these dissociative reversible resins move from a stiff thermoset network to a loosed state assimilable to a melted thermoplastic. Inside the dissociative group, the use of the Diels–Alder chemistry must be considerably widespread to generate switchable crosslinks by introducing intermediary maleimide and furan groups present on the polymer chains [9,10,11,12]. D-A crosslinking possesses the advantages of requiring mild reaction conditions, forming few by-products, and having no catalyst requirements. Moreover, the retro D-A reaction usually presents a fast kinetic (near to 10 min), which allows a quick reorganization of the network, and the temperature ranges of disengagement (from 90 to 160 °C) and rearrangement (around 60 °C) [9,13,14] are relatively moderate. The main disadvantage of using D-A bonds is the slowness of their rearrangement, which takes several hours.

In the same way as conventional polymers, conductive fillers can be added to the thermo-reversible structures to generate electroactive and multifunctional polymers [15,16] with self-healing, formability, and shape memory capabilities. Once the dispersion of these reinforcements is adequate in terms of quantity and distribution, a conductive network runs throughout the material, providing electrical pathways and modifying the insulating behavior of the resin to form a moderately electrically conductive material. Related to that change, these nanocomposites can be heated through the Joule effect by applying electric tension, triggering self-healing, thermoforming, or shape recovering. In this regard, the use of graphene [17] and graphene oxide [18] platelets was recently shown to be adequate for achieving electroactive switchable nanocomposites, in which the overcoming of a 1.5 wt.% of nanofillers leads to the initiation of partially conductive behavior, albeit with a non-ohmic response. The addition of graphene nanosheets hinders chain mobility and increases the capacity of shape fixing after applying a thermoforming process, although this restriction may obstruct shape memory effects after applying a new electrical current to trigger stress relaxation on the thermoformed structure [17,19]. Silver nanowires were also tested, with promising results for the development of self-healable composites due to their high conductivity [20,21]. In this regard, silver nanowire contents below 0.5 wt.% may be enough to reach the percolation threshold and find conductive networks [22], but the economic cost of using silver nanoparticles and possible future problems regarding the supply chain related to reaching peak silver production [23] may limit its use for industrial solutions. In this regard, carbon nanotubes (CNT) are also highlighted due to their combination of high aspect ratio and surface area as a particularly effective reinforcement, as they need notably low contents to overcome the percolation thresholds of the resins [24,25]. In addition, their cost has been significantly reduced in recent years due to their processing via CVD.

Despite the recent advances in thermo-reversible nanocomposites, this field is still evolving, and the research published about CNT nanocomposites made with D-A crosslinked polymeric matrices is scarce, especially for epoxy resins. In this regard, Willocq et al. [26] and Wang et al. [27] developed Diels–Alder crosslinked polyurethane nanocomposites reinforced with carbon nanotubes, reaching electrical healable materials through the activation of the retro D-A reaction via Joule heating. However, the CNT contents needed to achieve adequate electrical conductivity, i.e., from 1.5 wt.% to 5 wt.%, are considerably high. Handique and Dolui [28] and Lima et al. [29] also explore this field through the manufacturing of D-A epoxy and polyketone nanocomposites, respectively. The polyketone nanocomposites showed percolation thresholds similar to those of D-A polyurethanes to achieve electrical conductivity, i.e., higher than 1.5 wt.%. Meanwhile, the epoxy nanocomposites were manufactured with a 5 wt.% CNT content but were not characterized based on their electrical conductivity or any other thermo-electrical aspects. To the best of our knowledge, there are no previous works that analyzed the characterization of electrical and thermo-electrical properties of dissociative epoxy nanocomposites that included the influence of the content of Diels–Alder crosslinks on them, as well as the use of the electroactive capabilities to trigger self-healing through Joule heating in these specific nanocomposites.

According to the state of the art, we manufacture, in the present work, several nanocomposites with different molar contents of irreversible epoxy/amine bonds and reversible epoxy/D-A crosslinks (100/0, 40/60 and 0/100), as well as different contents of nanoreinforcement (0.1, 0.3, 0.5, 0.7, and 0.9 wt.%), are used to analyze the effect of the matrix on carbon nanotubes dispersion and the electrical and thermal properties of nanocomposites. The manufacturing method of the nanocomposites is based on previous works on Diels–Alder resins that achieved manufacturing success by dispensing with the use of solvents or high-viscosity oligomers [9] and non-reversible nanocomposites that achieved low percolation thresholds [25,30]. Although the addition of bismaleimide hinders the effectivity of the CNT dispersion process, insofar as bismaleimide is a solid mixed with CNT and epoxy resin during the dispersion step and aggregates to create zones with low CNT contents, the percolation thresholds of partial and fully reversible nanocomposites are clearly inferior to those previously observed in the bibliography [26,27,28,29], allowing ohmic behaviors and self-heating through the Joule effect. The fully reversible nanocomposites (ratio 1.0) have better CNT dispersions and electrical conductivities than the partially reversible nanocomposites, but they also show a higher brittleness that hinders their usability. Thus, the partially reversible nanocomposite (ratio 0.6) doped with a 0.7 wt.% of CNT was selected due to its more reasonable compendium of electrical conductivity and mechanical properties in order to evaluate the effect of CNT’s addition to mechanical and thermo-mechanical properties, as well as self-healing, proving the feasibility of repairing damage by applying an electric current.

## 2. Materials and Methods

### 2.1. Materials

The reagents used for the resins manufacturing were as follows: Bisphenol A diglycidyl ether (DGEBA), m-xylylenediamine (MXDA), furfurylamine (FA), and 1,1′-(Methylenedi-4,1-phenylene) bismaleimide (BMI). All these components were purchased from Merck (Darmstadt, Germany) and used as received. To nanoreinforce them, MWCNTs NC7000 from Nanocyl (Sambreville, Belgium) with average diameters of 9.5 nm were used.

### 2.2. Manufacturing

**Figure 1:**
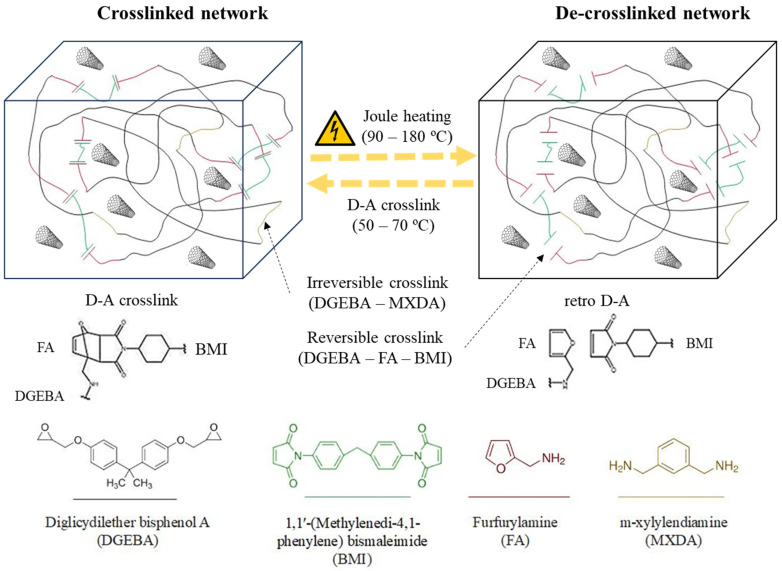
Scheme of electroactive and thermo-reversible structures of nanocomposites.

As the first step, CNTs were mixed with DGEBA and dispersed using a three-roll mini calander (Exakt 80E, Norderstedt, Germany) at ambient temperature, using a previously optimized procedure for determining roller rotation speeds (250 rpm) and gaps (from 120 to 15 µm) [25,30]. Once the nanoreinforcement was correctly incorporated into the epoxy monomer, BMI was added, and the mixture was heated up to 80 °C to further enable mechanical stirring and degassing for 15 min. After degassing, the reagents FA and MXDA were added and mixed, and the obtained mixtures were molded to obtain the nanocomposites. The curing cycles of these resins were set at a temperature of 100 °C for 6 h to generate covalent bonding, followed by the second stage at 60 °C for 12 h to generate the Diels–Alder (D-A) crosslinking, according to a method shown in a previous work [9]. The scheme of the polymeric network is shown below in Figure 1. To analyze the influence of polymer chemistry on the properties of nanocomposites, nanocomposites with a 0.6 D-A ratio and a 1.0 D-A ratio were manufactured. The adjustment of the resin compositions was carried out according to the following equations:

(1) $$m_{FA}=\frac{m_{DGEBA}\cdot {Neq}_{DGEBA}}{{Mw}_{DGEBA}}\cdot \frac{{Mw}_{FA}}{{Neq}_{FA}}\cdot {DA}_{ratio}$$

(2) $$m_{BMI}=\frac{m_{FA}}{{Mw}_{FA}}\cdot {Mw}_{BMI}\cdot \frac{1}{2}$$

(3) $$m_{MXDA}=\frac{m_{DGEBA}\cdot {Neq}_{DGEBA}}{{Mw}_{DGEBA}}\cdot \frac{{Mw}_{MXDA}}{{Neq}_{MXDA}}\cdot (1-{DA}_{ratio})$$

Moreover, DGEBA-MXDA samples (0 D-A ratio) were manufactured and tested to set a reference for the analysis. To create these conventional and irreversible crosslinked nanocomposites, CNTs were dispersed using the same method, and then the resin was degassed at 80 °C for 15 min and cured at 60 °C for 30 min, after this first step, at 100 °C for 6 h. The thermal curing treatments were previously optimized [9], confirming the maximum conversion reached for the different implied chemical reactions.

### 2.3. Characterization

CNT dispersions in non-cured epoxy mixtures were analyzed using a Leica (Wetzlar, Germany) high-transmission optical microscope (TOM), equipped with a Nikon Coolpix 990 camera. Meanwhile, carbon nanotube dispersion in the cured nanocomposites was observed through electron microscopy. The samples were cryogenically fractured using liquid nitrogen, coated with a thin layer of gold, and observed via Field Emission Gun Scanning Electron Microscopy (FEG-SEM, Nova NanoSEM 230, FEI, Hillsboro, OR, USA).

DC volume conductivity tests were carried out using samples whose dimensions were 10 × 10 × 1 mm^3^. Electrical conductivities were determined through the electrical resistance of the specimens, defined as the slope of the current-voltage (I–V) curves of the conductivity tests. In this regard, the voltage ranges were set as 0–100 V for low-conductivity samples and 0–10 V for high-conductivity samples. These tests were performed in a Keithley 2410 multimeter sourced from Keithley Instruments (Cleveland, OH, USA).

Thermoelectrical properties were determined via resistive heating. In this experiment, samples were subjected to a varying applied voltage, while temperature change was measured via a thermographical technique using a FLIR (Wilsonville, OR, USA) E50 camera. The sample dimensions were 37 × 12.7 × 1.8 mm^3^, and the electrical currents were applied by winding the ends of the samples with copper wires and adding silver paint to ensure proper electrical contact.

Differential Scanning Calorimetry (DSC) tests were carried out using a DSC25 device sourced from TA Instruments (New Castle, DE, USA) by setting a ramp from 30 to 240 °C with a 10 °C/min heating rate.

Thermomechanical properties were measured through Dynamical Thermomechanical Analysis (DTMA) with a Q800 from TA Instruments. Tests were carried out in single-cantilever mode by applying a constant strain of 0.1% and a frequency of 1 Hz. Thermomechanical data were collected in a temperature range from 30 to 200 °C, with a heating rate of 2 °C/min.

Flexure tests were carried out at ambient temperature using a universal testing machine Zwick Z100 (Ulm, Germany) in three-point bending mode using a 500 N load cell with a crosshead speed of 1.5 mm min^−1^ via ASTM D790 [31]. The fracture surfaces were sputtered with a thin layer of gold and observed via Scanning Electron Microscopy (SEM) using a Hitachi S3400N (Tokyo, Japan).

Remendability was characterized by first promoting controlled damage with a knife and analyzing the crack before and after the healing thermal treatment using an optical 3D profilometer from Zeta Instruments (Phoenix, AZ, USA).

## 3. Results

### 3.1. Analysis of Nanocomposite Manufacturing and Thermo-Electrical Properties

The resins were synthesized using a two stages process. Firstly, CNT were dispersed in the DGEBA resin, and all reagents were then mixed and heated at a temperature of 100 °C for 6 h to initiate the curing reaction among oxirane rings and amines. Higher curing temperatures were discarded to prevent furfurylamine volatilization, Michael reaction (formation of amine-maleimide irreversible bonds), and bismaleimide homopolymerization [9]. After that, the partially and fully reversible nanocomposites were heated at 60 °C for 12 h to generate the Diels–Alder bonds and complete the crosslinked network. After this stage, a reversible nanocomposite network that could change from a rigid and crosslinked structure to a loose and healable one (Figure 1) was achieved. Crosslink switching could be reached via either convective or electrical heating.

**Figure 2:**
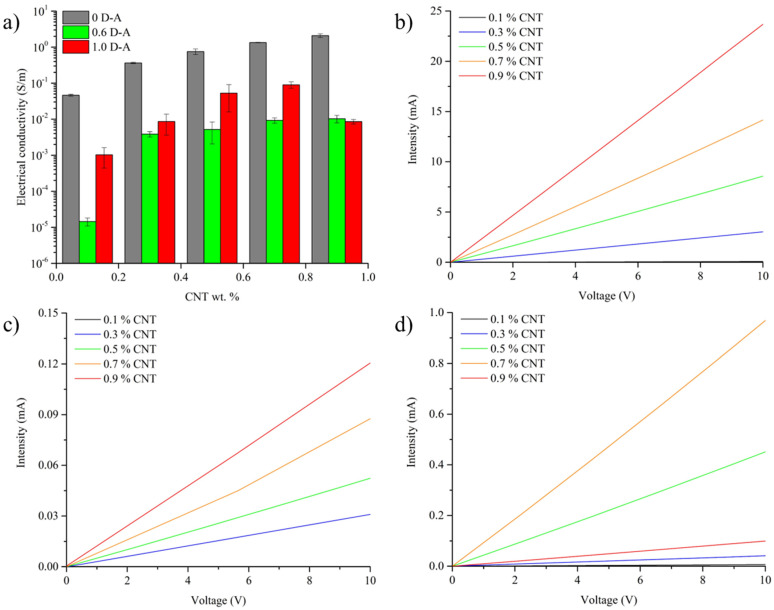
Electrical conductivities of nanocomposites (**a**) and comparison between I-V curves of 0 D-A (**b**), 0.6 D-A (**c**), and 1.0 D-A ratio (**d**) nanocomposites.

As shown in Figure 2a, the resin without Diels–Alder bonds (0 D-A ratio) shows a variation in electrical conductivity with the content of carbon nanotubes comparable to other conventional epoxy resins, with the percolation threshold reached by adding 0.1 wt.% of CNT [19,24,25]. This behavior varies in the resins with Diels–Alder bonds, which have lower electrical conductivity. However, the nanocomposites, regardless of their chemical compositions and nanotube contents (except in the case of the 0.6 D-A ratio nanocomposite doped with a 0.1 wt.% of CNT, which is below the percolation threshold and non-conductive), show ohmic behaviors (Figure 2b–d). In the case of the partially reversible resin (0.6 D-A ratio), the percolation threshold is not reached until adding 0.3 wt.% of CNT. On the other hand, the fully reversible nanocomposites’ (1.0 D-A ratio) results are somewhat better. The conductivity in this resin increases significantly with the addition of nanotubes up to 0.7% by weight, reaching an average conductivity of 0.09 S/m. However, the addition of higher contents up to a 0.9 wt.% in the 1.0 D-A resin leads to the formation of CNT aggregates and reduces the electrical conductivity of the nanocomposite.

**Figure 3:**
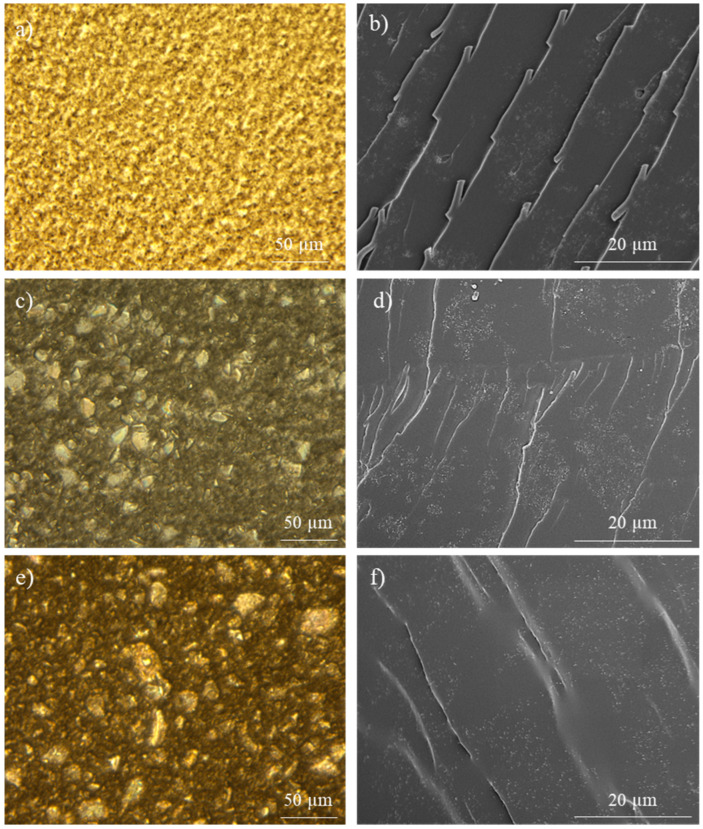
Observations of CNT dispersions of nanocomposites doped with 0.7 CNT wt.% via optic microscopy after calendaring, as well as in FEG-SEM after being cured: 0% D-A ratio (**a**,**b**), 0.6 D-A ratio (**c**,**d**), and 1.0 D-A ratio (**e**,**f**).

**Figure 4:**
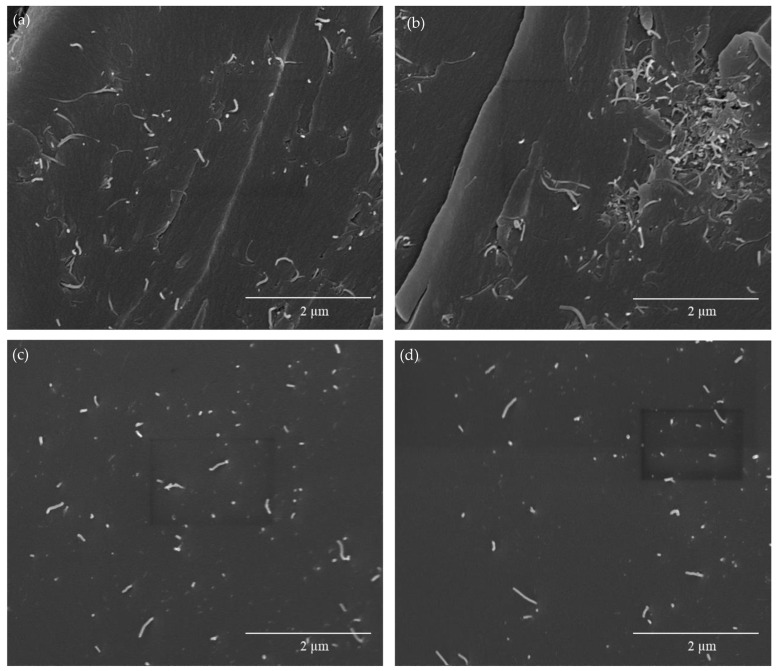
FEG-SEM observations at 50,000× *g* of the 0 D-A ratio nanocomposite (**a**,**b**) and the 1.0 D-A ratio nanocomposite (**c**,**d**) doped with 0.7 CNT wt.%.

**Figure A1:**
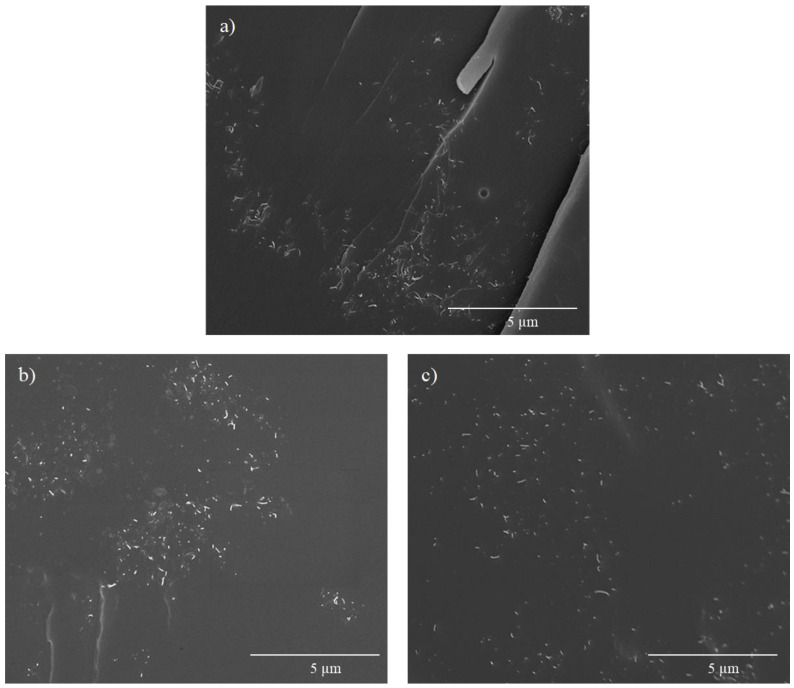
FEG-SEM observations of 0 D-A (**a**), 0.6 D-A (**b**), and 1.0 D-A ratio (**c**) nanocomposites doped with 0.7 CNT wt.% at 20,000×.

In order to explain these results, the morphologies of non-cured mixtures and final nanocomposites were studied. Fresh resins were observed via an optical microscope, and cured nanocomposites were observed via SEM and FEG-SEM. The addition of bismaleimide to create reversible networks entails a significant presence of solid agglomerates of this reagent on dispersions, which reduce the homogeneity of CNT distribution (Figure 3a,c,e) after calendaring. Nonetheless, BMI is dissolved after amine mixing and during the first moments of nanocomposite curing before the gel time. FEG-SEM observations of cured nanocomposites (Figure 3b,d,f) reveal clear differences in the distribution of CNT depending on the matrix. The 0 D-A ratio resin shows a homogeneous dispersion with a certain presence of the small agglomerates, which are close to each other. The 0.6 D-A ratio nanocomposite shows a distribution of nanotubes in aggregates, many of them isolated from each other, which agrees with both the lower values of electrical conductivity for these materials [25] and the observations of fresh resin via optic microscopy. In contrast, the cured 1.0 D-A ratio resin shows a more homogeneous dispersion that provides better electrical behavior compared to 0.6 D-A nanocomposites. The curing reaction of the 1.0 D-A ratio resin is slower than that of the 0.6 D-A ratio resin [9], and consequently, the gelation time is longer, which also allots more time for BMI dissolution and might allow certain enhancement of the homogenization of the resin and CNTs mixtures in these nanocomposites. In this regard, the absence of small and unconnected agglomerates can be observed. Compared to the 0 D-A ratio nanocomposites, the distances between nanotubes observed at high magnifications in the 1.0 ratio nanocomposite are higher, something that could explain why these nanocomposites have lower electrical conductivities than those of the 0 ratio nanocomposites (Figure 4) [32,33]. A comparison including the 0.6 D-A ratio nanocomposite at high magnifications is included in Figure A1.

Beyond the state of nanoreinforcement distribution in nanocomposites, the possible influence of matrix-reinforcement interfaces must also be considered. Recent modeling has shown that bismaleimide generates a stronger interfacial interaction with CNT than those exerted by epoxy monomers [34]. Increased CNT encapsulation in reversible resins caused by bismaleimide contents may hinder electron transmission through direct contact, something that could also contribute to the decrease in their electrical conductivities compared to 0 D-A ratio nanocomposites.

The electrical conductivities of the different nanocomposites are reflected in their thermoelectrical responses, since their Joule heating can be modeled, ideally, according to the following equation:

Q = I^2^·R·t = m·C_p_·ΔT (4)

**Figure 5:**
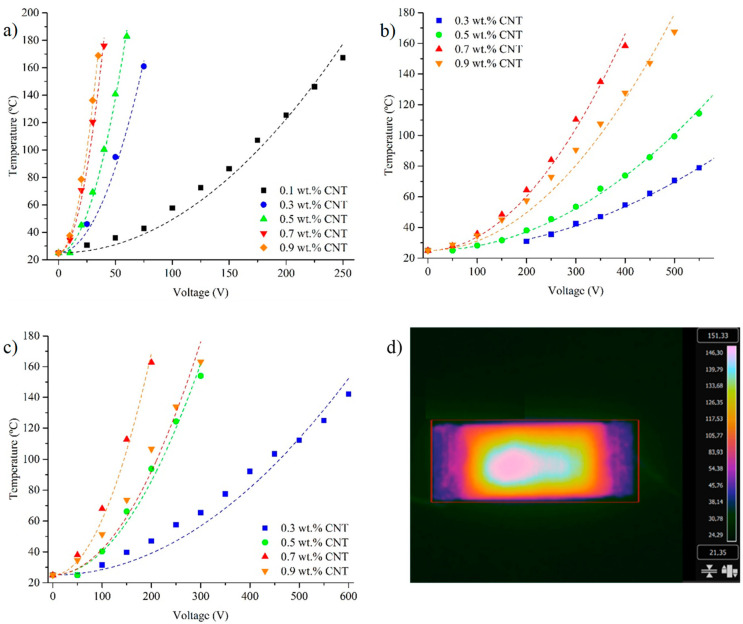
Comparative Joule heating levels for the nanocomposites: 0 D-A ratio nanocomposites (**a**), 0.6 D-A ratio (**b**), and 1.0 D-A ratio (**c**). Thermographic picture of Joule heating (**d**).

The 0 D-A ratio nanocomposites, due to their more effective CNT distributions and higher electrical conductivities, need considerably low voltages to reach the temperatures required for the full retro D-A reaction (150–170 °C) that are settled as targets for 0.6 and 1.0 D-A nanocomposites. In this regard, it can be seen in Figure 5a that the addition of CNT up to a 0.3 wt.% completely overcomes the percolation threshold of the 0 D-A resin, and further nanoreinforcement additions do not offer notable improvements in thermoelectrical response, especially above 0.7 wt.%. This behavior changes in both partially (0.6 D-A) and fully (1.0 D-A) reversible resins (Figure 5b,c). The addition of CNT up to a 0.3 wt.% is needed to reach the percolation threshold, as shown in Figure 2, and achieve a perceptible thermoelectrical response, although is not sufficient to reach the temperatures required for full D-A network disengagement.

**Figure A2:**
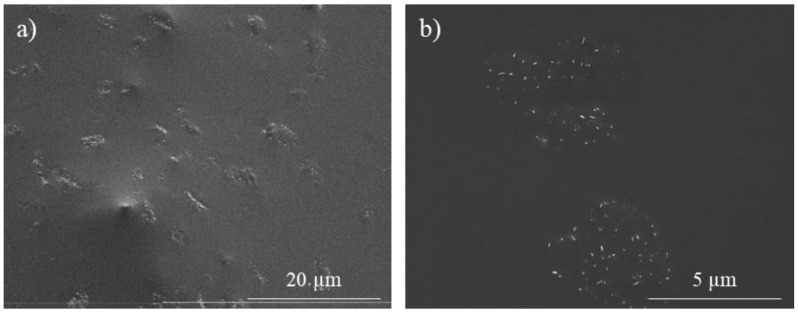
FEG-SEM observations of 1.0 D-A ratio nanocomposites doped with 0.9 CNT wt.% at 5000× (**a**) and 20,000× (**b**).

In both resins, the best results are obtained by adding a 0.7 wt.% of CNT when the percolation thresholds are fully surpassed, but the amounts of nanoparticles are not enough to reduce the quality of the dispersions via regrouping into large aggregates, as happens in the nanocomposites doped with a 0.9 wt.% of CNT (shown in Figure A2).

It is worth noting the high correlation between the temperatures reached using the samples and the voltages applied to the ideal quadratic model defined by Equation (4). The R^2^ values in 1.0 D-A ratio resins doped with 0.3% and 0.9% wt.% of CNT, which are the worse cases, remain at 0.96. Meanwhile, the other samples analyzed present R^2^ values between 0.98 and 0.99. These results corroborate the ohmic behavior of the nanocomposites, even at high temperatures. In this regard, the disengagement of the reversible network via retro Diels–Alder reaction does not seem to have appreciable effects on the electrical conductivities of nanocomposites.

**Figure 6:**
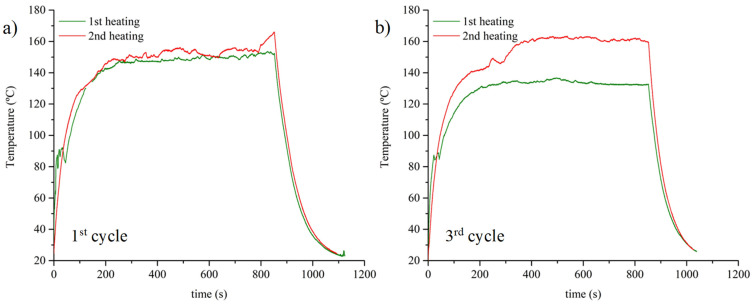
Joule heating curves of 0.6 D-A nanocomposite doped with a 0.7 wt.% of CNT: first cycle of heating after the manufacturing (**a**) and curves from the third cycle of heating and D-A recovery (**b**).

The application of several cycles of Joule heating and Diels–Alder recovery reveals some interesting results, as shown in Figure 6. These repeated tests were realized using the 0.6 D-A nanocomposite doped with 0.7 wt.% of CNT, discarding the 1.0 D-A resin to avoid possible effects related to its loss of form and partial melting when high temperatures are maintained for a certain period of time. During the first heating ramp occurring after the manufacturing of the samples, the nanocomposite shows quick heating from ambient temperature to approximately 90 °C. Then, the progression is sharply interrupted, and it was observed that the temperature oscillates from 90 to 80 °C for 20 to 30 s before increasing again up to the stationary state. This oscillation of temperatures near to 90 °C coincides with the initiation of the retro D-A reaction, which is an endothermic phenomenon that can be started at this same temperature, as mentioned in the introduction. The sample is maintained in a stationary state for 10 min to complete the D-A network disengagement. During the cooling down period, we did not observe any particular behavior; unlike the retro D-A reaction, the formation of D-A bonds is much slower and cannot happen in significant amounts during the temperature decrease. Once the sample is cooled down to an ambient temperature, the Joule heating is repeated without rebuilding the D-A network. From ambient temperature to 90 °C, the second heating stage is somewhat less intense despite applying the same electrical power. This difference might indicate that the network disengagement could increase the specific heat of the polymer network, in a manner similar to what happens when a thermoset passes from a glassy to a rubbery state [35]. The second heating stage does not show any oscillation of temperatures around 90 °C, insofar as the endothermic retro D-A reaction cannot happen, and a progressive increase in temperatures up to the stationary state is observed. In this regard, the samples reach higher temperatures in comparison to the first heating, probably caused by the endothermic disengagements of -exo D-A isomers, which happen at temperatures above 130 °C.

After the second heating stage, the samples are placed in an oven at 60 °C for 12 h to generate D-A crosslinks. The reconstruction of the crosslinked network makes the material’s response to Joule heating reversible: after three cycles of Joule heating and D-A reaction, the nanocomposites still show similar behaviors, as shown in Figure 6b. The first heating stage is marked by a sharp increase in temperature from ambient temperature to 90 °C that is interrupted by the retro D-A reaction. During some seconds, the temperature oscillates between 80 and 90 °C before rising again up to the stationary state. After cooling down, the second heating stage shows a slightly lower intensity up to 90 °C, but it does not oscillate, reaching higher temperatures in the stationary state.

**Figure 7:**
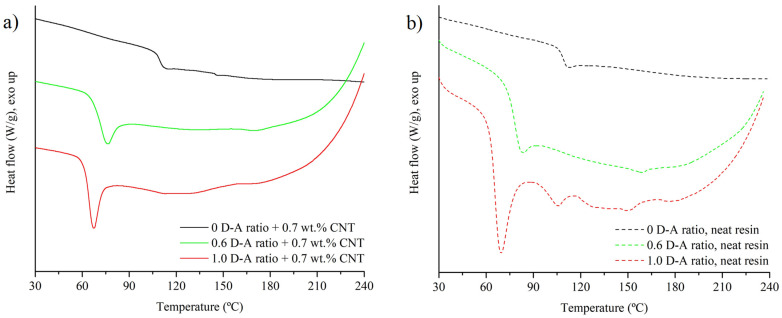
DSC scans of 0, 0.6, and 1.0 D-A ratio nanocomposites reinforced with 0.7 wt.% of CNT (**a**) and DSC scans of 0, 0.6, and 1.0 D-A ratio neat resins (**b**).

Finally, to check if the addition of CNT influences the resins’ curing kinetics or induces side reactions, the cured samples were analyzed via DSC scans from ambient temperature to 240 °C. Figure 7 shows a comparison between the DSC scans of the nanocomposites doped with a 0.7 wt.% of CNT (Figure 7a) and the DSC scans of the neat resins (Figure 7b). The thermal behavior of the cured neat resins was determined in a previous work [9], observing, in summary, three main characteristics:

1. A notable decrease in T_g_ induced via the substitution of the DGEBA-MXDA chain with D-A crosslinks, passing from 108 °C in the 0 D-A ratio sample to 64.4 °C in the 1.0 D-A ratio resin.
2. An endothermic region, which corresponds to the retro D-A reaction, appears from 90 to 180 °C. At lower temperatures, i.e., closer to 100 °C, the endothermic peaks correspond to -endo D-A isomers’ disengagement; above 130 °C, the endothermic peaks are associated with -exo D-A isomers’ dissociation [12].
3. An exothermic region above 190 °C appears due to BMI homopolymerization [36]. The apparition of this reaction confirms that no side irreversible reactions, such as Michael addition, happen to a significant extent during resin manufacturing and curing.

As observed in Figure 7 and Table 1, the incorporation of high loads of CNT does not introduce notable changes into the thermal response of the samples, confirming that they do not interfere in the curing cycle of the nanocomposites. The glass transition temperatures of neat resins and nanocomposites are similar, observing slight increases for nanocomposites with 0.6 and 1.0 D-A crosslink ratios due to the stiffening of reversible resins at the CNT location in the free volume of matrix. The glass transition of D-A nanocomposites are accompanied by enthalpy relaxation peaks: the storage of these materials at 60 °C for 12 h after curing, to create the D-A network, induces significant thermal aging, since this reaction exposes the materials to sub-T_g_ for long times and causes a non-equilibrium state in resin networks [37]. The effect of CNT dispersion is evident, insofar as the 0.6 D-A nanocomposite exhibits a higher peak compared to the neat resin, whereas that of the 1.0 D-A nanocomposite shows the opposite trend. CNTs, whether functionalized or not, interact effectively with the matrices, if they are correctly dispersed, and protect them from physical aging [38], but this influence can be lost if the amounts added are too high and form aggregates [39]. The results observed for DSC scans are aligned with previous FEGSEM observations, in which the 1.0 D-A nanocomposite shows a fine and homogeneous dispersion; meanwhile, the 0.6 D-A nanocomposite has numerous aggregates that could favor thermal aging.

Above the glass transition temperature, the nanocomposites show the expected endothermic region from 90 to 180 °C associated with the retro D-A reaction. Nevertheless, the enthalpies of retro D-A reaction obtained in both reversible nanocomposites are around 40% to 50% higher than those observed in neat resins. The relatively high content of CNT contributes to generating an expanded D-A network in these nanocomposites. 

Carbon nanotubes, such as other carbon nanostructures like graphene or fullerenes, can act as both diene and dienophile and build D-A adducts with free furan and maleimide groups through their unsaturated C=C bonds [40,41,42,43], giving higher crosslink densities at lower temperatures that could promote the slight increases in T_g_ observed. After the retro D-A reaction, the free maleimide groups homopolymerize through an exothermic reaction when the temperature exceeds 190–200 °C. The tendencies observed in nanocomposites at these temperatures are similar to the thermal responses of neat resins, which confirms that no side reactions that could impede the D-A network formation happen during the manufacturing of nanocomposites.

### 3.2. Mechanical and Thermomechanical Characterization of the 0.6 D-A Resin and Nanocomposite

Before carrying out an accurate analysis of the self-healing capabilities of the electroactive D-A nanocomposites, it is convenient to evaluate their mechanical and thermomechanical properties. In this regard, both properties were previously studied on neat resins with different D-A ratios ranging from 0 to 1.0 via DMTA and three-point-bending tests [9]. The results for the 0, 0.6, and 1.0 D-A ratio resins are shown in Table A1. In general, it was found that the substitution of irreversible covalent DGEBA-MXDA bonds by reversible D-A chains led to a gain in recyclability and repairability, in exchange for a notable loss of thermal and mechanical properties. Apart from the decrease in T_g_ in the DSC analysis shown previously in Table 1, the increase in the D-A crosslink ratio induces higher brittleness in epoxy resins and reduces their mechanical strength. This aspect is especially critical in the 1.0 D-A ratio resin, whose lack of strength (7.2 ± 0.6 MPa) and brittle structure reduce its practical utility. Even more, this negative effect can be worsened if it is needed to add high amounts of nanoreinforcements to achieve enough electrical conductivity, something that considerably increases the viscosity before curing and hinders its processing. The poor properties observed in neat 1.0 D-A resin are complemented by the nanocomposites fractographies observed in FEG-SEM (Figure 3), which show smooth surfaces proper of quick crack propagation in highly brittle materials. These results rule out the 1.0 D-A ratio resin as a useful candidate for the synthesis of thermoformable and repairable nanocomposites. On the other hand, the 0.6 D-A ratio resin presents an acceptable compromise in terms of the properties that make it potentially valid for the development of thermoformable, recyclable, and self-healable electroactive nanocomposites. The glass transition temperature (91 °C) and the strength (75 MPa) of this resin are still considerable, and, moreover, the introduction of partial contents of D-A chains increased the stiffness considerably compared to a DGEBA-MXDA resin. Moreover, the 0.6 D-A resin showed excellent recyclability and shape memory due to its network relaxation.

**Figure 8:**
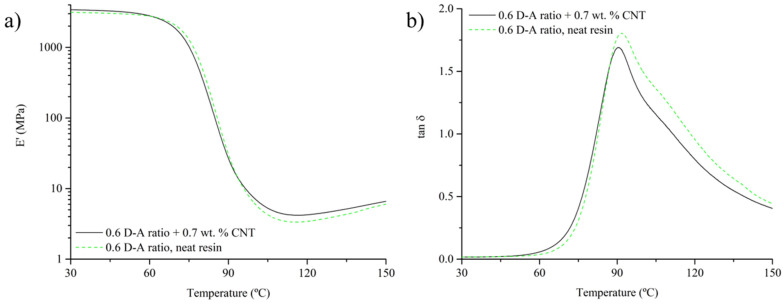
Comparison between thermomechanical properties of a 0.6 D-A ratio nanocomposite reinforced with a 0.7 wt.% of CNT and a 0.6 D-A ratio neat resin: storage modulus (**a**) and tan delta (**b**) vs. temperature curves.

The addition of CNT up to a 0.7 wt.% on 0.6 D-A resin increases the polymeric network stiffness. The presence of CNTs introduces interactions such as the formation of D-A adducts with FA and BMI observed in DSC analysis, as well as physical entanglements related to free volume occupation, that could restrict the segment mobility of the polymer chains [44]. Thus, the storage modulus of the nanocomposite is higher compared to the neat resin both in glassy and rubbery states, as shown in Figure 8a.

However, in terms of thermal resistance, the effect of CNT is not remarkable and does not affect the glass transition temperature of the nanocomposite measured through the maximum value of tan δ in DMTA (see Figure 8b). The nanocomposite shows a T_g_ of 91.0 ± 1.0 °C, which falls within the temperature range observed in the neat resin (91.1 ± 1.5 °C). 

**Figure 9:**
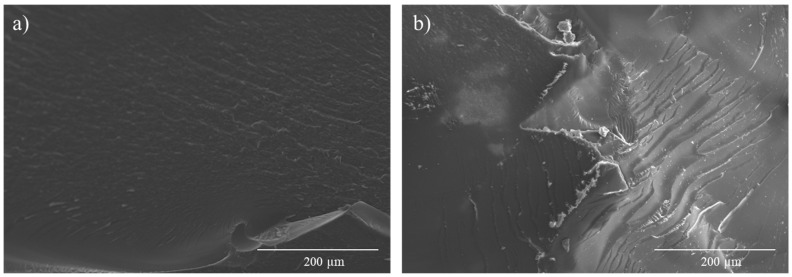
Comparison between fracture surfaces from three-point-bending tests of 0.6 D-A ratio reinforced with a 0.7 wt.% of CNT (**a**) and a 0.6 D-A ratio neat resin (**b**).

Otherwise, the 0.6 D-A ratio nanocomposite shows a certain decrease in mechanical properties compared to the neat resin, according to the results of the three-point-bending tests shown in Table 2. The reasons behind this loss of properties are varied. Firstly, the addition of high-volume contents of CNT, as this case, implies a remarkable rise in the viscosity of the resin, which can reach two orders of magnitude higher [45]. Secondly, degassing was carried out after calendaring, keeping air entrapped within the resin prior to the curing step. As a result, the cured nanocomposite may have some micro voids that can depauperate the mechanical response. Other important factors are the CNT distribution by aggregates, as observed in FEGSEM images previously shown in 0.6 D-A nanocomposites (Figure 3), which can favor crack propagation [46], and the thermal aging suffered by the nanocomposites during the D-A reaction, as previously mentioned in the DSC analysis. Nevertheless, the mechanical strength is still considerable if the data are compared to those of other D-A reversible epoxy nanocomposites [47], and the fracture surfaces of nanocomposite (Figure 9a) and neat resin (Figure 9b) present similar morphologies. In summary, despite the loss of mechanical properties, the development of a usable, electroactive, and partially thermo-reversible resin is still achievable according to the results.

The CNT used as reinforcements (NC7000) were selected in order to use standard materials, maintaining the idea of achieving the greatest possible manufacturing simplicity; this is something that is one of the main weaknesses in the development of epoxy resins with Diels–Alder bonds in research published to date. Nonetheless, the results indicate that dispersions and CNT-epoxy interactions can be improved in future developments to obtain enhanced smart nanocomposites. Several studies address these aspects through nanoarchitectonic approaches [48]. In this regard, the functionalization of CNT with amine groups could be an interesting pathway, as it was demonstrated in previous studies to be an adequate method for increasing the mechanical properties of nanocomposites [49]. In the case of the Diels–Alder nanocomposites in the present work, the amines’ addition must be carefully balanced to avoid the Michael reaction, so the adoption of these kind of pathways would be somewhat risky. The reduction in the furfurylamine content would be considered to be compensation, insofar as functionalized CNT can also act as dienes and create D-A bonds with unreacted maleimides, as indicated in other studies [42]. Moreover, amine functionalization could also help to maintain CNT dispersion and reduce re-agglomeration via surface wrapping. However, this effect could have both positive and negative results: the improvements in CNT dispersions could benefit the electric transmission, but, on the other hand, CNT insulation via polymeric wrapping could increase the potential barrier. Other possibilities would involve optimizations of the mixing and dispersion methods. In any case, the possible pathways for improvement point to further exhaustive analysis, either in terms of nanoreinforcement selection or manufacturing techniques.

### 3.3. Smart Abilities: Thermally and Thermoelectrically Stimulated Crack-Healing and the Shape Memory of the 0.6 D-A Resin Nanocomposite Doped with 0.7 wt.% of CNT

**Figure 10:**
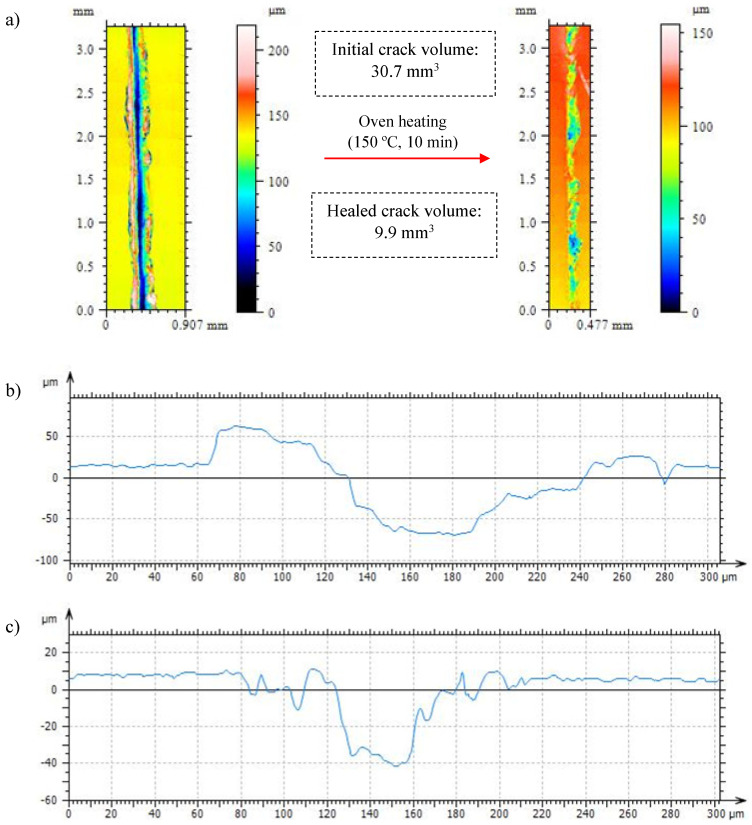
Plan view and volume measuring for the crack created on the neat 0.6 D-A resin before and after healing (**a**) and crack profiles before (**b**) and after healing (**c**) through oven heating.

**Figure 11:**
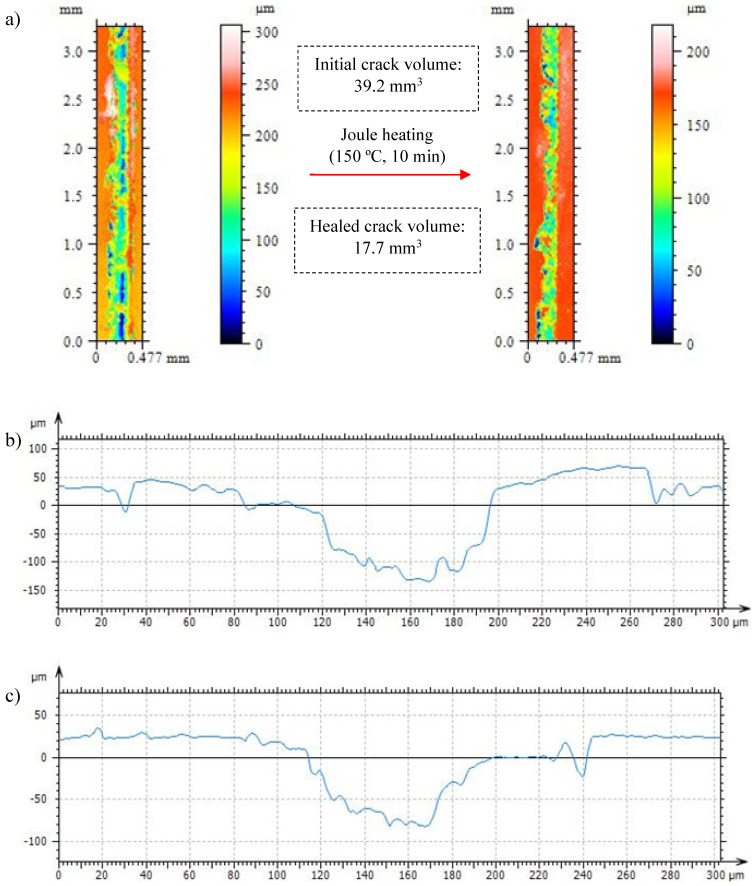
Plan view and volume measuring for the crack created on the 0.6 D-A nanocomposite doped with a 0.7 wt.% of CNT before and after healing (**a**) and crack profiles before (**b**) and after healing (**c**) through Joule heating.

Therefore, the healing capabilities of both neat resin and nanocomposite are evaluated to analyze the autonomous damage healing of the nanocomposite via electric stimulus compared to the convective heating of the neat resin in an oven. Firstly, both materials were damage when using a knife, creating a crack with a 100 to 150 µm of depth. The characterization of the cracks and their healing is shown in Figure 10 and Figure 11. Crack healing involves a physical response of the polymer by allowing chain diffusion into the damaged areas [14]. This phenomenon is achieved by heating the resins up to 150 °C to trigger the retro Diels–Alder reaction and the segmental mobility of those parts of the networks composed of reversible bonds. The softening of the network enables the partial sealing of damage: crack’ depths and volumes decrease considerably after maintaining the resins at 150 °C for 10 min. According to its thermo-mechanical properties, the neat resin shows enhanced healing compared to the nanocomposite due to its higher network softening and chain mobility. The disengagement of the Diels–Alder bonds and the structural relaxation in neat resin reduce the crack volume up to a 68%. Despite Joule heating being a fast and internal heating-based pathway that could help to homogenize the temperatures across the crack’s surface, the volume of crack reduction in the nanocomposite reaches 55%. The higher stiffness of the nanocomposite in a rubbery state due to CNT presence restricts chain mobility and hinders the viscoelastic flow that refills the crack volume once the material is heated up to enable the retro D-A reaction. The lower crack-healing capacity of the nanocomposite compared to the neat resin, caused by the lesser network mobility, is parallel to the decreases observed in the bibliography regarding other nanocomposite properties, such as shape memory [19].

Nonetheless, the addition of functionalities such as electric conductivity and resistive heating to covalent adaptable networks is a promising route, insofar as this kind of nanocomposite can lead to new autonomous structures that can be diagnosed and repaired in an automated and remote way.

**Figure 12:**
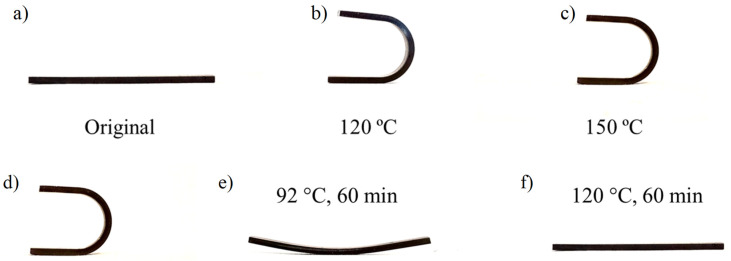
Shape fixing (**a**–**c**) and shape recovery (**d**–**f**) of a 0.6 D-A nanocomposite doped with a 0.7 wt.% of CNT. Shape fixing: initial shape (**a**) and U-shape thermoforming at 120 (**b**) and 150 °C (**c**). Shape recovery: initial shape (**d**) and shape recovery at 92 °C (**b**) and 120 °C for 1 h.

Finally, other functionalities of the reversible nanocomposites are their ability to shape thermoconforming and recovery. Figure 12 shows the related results. Considering the T_g_ measurement to be 92 °C, the thermoconforming process must be carried out at higher temperatures due to mobility restrictions of CNT. The minimum required shaping temperature is 120 °C. No great differences are observed at higher temperatures. 

For shape recovery, the shaped geometry can return to its initial geometry at temperatures higher than the T_g_, obtaining a shape memory efficiency close to 100%. 

The optimized nanocomposite shows a moderately high T_g_ of around 91 °C and high stiffness combined with some smart functionalities, such as electrically triggered self-healing and shape memory. This compendium of properties makes them a suitable material for use in applications in which stiffness at low temperatures combined with self-healing, formability shape memory, and flexibility at higher temperatures can be advantageous, such as protective coatings [50] or certain applications of soft robotics [51], discarding specific fields like biomedicine [52] due to the high temperatures needed for network disengagement. Looking at other aspects like economic feasibility, the reagents used for the synthesis, such as DGEBA and amines, are common, and bismaleimide prices are not excessively high [53]. Even more, the manufacturing method does not need solvents and has no differences compared to other manufacturing routes for conventional thermosets and nanocomposites without reversible bonds. The results, in summary, represent an advance toward increasing the economic competitiveness of self-healable and smart nanocomposites.

## 4. Conclusions

The manufacturing of electroactive and thermo-reversible epoxy nanocomposites with D-A bonds is realized using a simplified methodology that dispenses with the use of solvents or manufacturing in several stages via the manipulation of high-viscous intermediate oligomers. The reversible nanocomposites achieve ohmic behaviors and reasonable electrical conductivities with CNT contents below 1.0 wt.%, with results that contrast with the high CNT contents usually being added to dissociative nanocomposites. According to the results, the following conclusions can be settled:

1. The addition of BMI and its formation of aggregates hinder the effectiveness of CNT dispersion and make difficult direct contact between them. Thus, D-A nanocomposites show lower electrical conductivities, but they have ohmic behaviors and predictable heating through the Joule effect.
2. The addition of CNT up to 0.7 wt.% allows the optimum thermoelectric responses of D-A nanocomposites, which are able to reach a range of temperatures between 150 and 170 °C and trigger reversible network disengagement through retro D-A reaction.
3. The 1.0 D-A ratio nanocomposites have a high brittle behavior with smooth fracture surfaces that reduce their usability. Meanwhile, the 0.6 D-A ratio nanocomposites show a more balanced compendium of properties despite their lower electrical conductivity.
4. CNT addition reduces chain mobility at high temperatures. Nonetheless, a 0.6 D-A ratio nanocomposite can heal cracks via Joule heating and reduce its original volume by more than 50%.
5. The shape fixing and shape recovery efficiency of a 0.6 D-A ratio nanocomposite is close to 100 °C when the thermoconforming and shape recovery temperatures are higher than the T_g_, i.e., approximately T_g_ + 20 °C.

## Figures and Tables

**Table 1:** Comparison between the glass transition temperature and enthalpy of the retro D-A reaction among neat resins and nanocomposites doped with 0.7 wt.% of CNT.

	T_g_ (°C)	ΔH_rD-A_ (J/g)
0 D-A ratio + 0.7 wt.% CNT	107.7	-
0.6 D-A ratio + 0.7 wt.% CNT	69.8	6.5
1.0 D-A ratio + 0.7 wt.% CNT	66.7	12.2
0 D-A ratio, neat resin	108	-
0.6 D-A ratio, neat resin	67.6	4.7
1.0 D-A ratio, neat resin	64.4	8.2

**Table 2:** Comparison between the mechanical and thermomechanical properties of the neat 0.6 D-A resin and the 0.6 D-A nanocomposite doped with a 0.7 CNT wt.%.

	0.6 D-A Ratio Neat Resin	0.6 D-A Ratio + 0.7 CNT wt.%
E’_G, 30 °C_ (MPa)	3129 ± 233	3336 ± 119
E’_R, min_ (MPa)	3.3 ± 1.3	4.9 ± 1.0
T_g_ (°C)	91.1 ± 1.5	91.0 ± 1.0
E’_30 °C_/E’_Tg_	133–168	112–136
E’_G, 30 °C_/E’_R, min_	1070–1470	570–820
σ _flex_ (MPa)	75.0 ± 11.8	33.9 ± 5.4
ε _u, flex_ (%)	2.5 ± 0.5	1.4 ± 0.2

## Data Availability

Data are contained within the article.

## Appendix A

**Table A1:** Comparison of thermo-mechanical and flexural properties of 0 D-A, 0.6 D-A, and 1.0 D-A neat resins.

	0 D-A Ratio	0.6 D-A Ratio	1.0 D-A Ratio
E’_30 °C_ (MPa)	2656 ± 146	3129 ± 233	2045 ± 487
E’_min_ (MPa)	21.5 ± 1.6	3.3 ± 1.3	-
T_g_ (°C)	126.5 ± 0.3	91.1 ± 1.5	80.3 ± 1.9
σ _flex_ (MPa)	152.2 ± 13.9	75.0 ± 11.8	7.2 ± 0.6
E _flex_ (MPa)	3097.3 ± 224.0	4503.9 ± 246.5	2364.4 ± 245.0
ε _flex_ (%)	5.9 ± 1.0	2.5 ± 0.5	0.6 ± 0.1
